# A Low-Cost Sensor Buoy System for Monitoring Shallow Marine Environments

**DOI:** 10.3390/s120709613

**Published:** 2012-07-16

**Authors:** Cristina Albaladejo, Fulgencio Soto, Roque Torres, Pedro Sánchez, Juan A. López

**Affiliations:** DSIE, Technical University of Cartagena, Campus Muralla del Mar s/n, Cartagena E-30202, Spain; E-Mails: cristina.albaladejo@upct.es (C.A.); pencho.soto@upct.es (F.S.); roque.torres@upct.es (R.T.); jantonio.lopez@upct.es (J.A.L.)

**Keywords:** marine sensor system, sensor buoy, oceanography, remote monitoring

## Abstract

Monitoring of marine ecosystems is essential to identify the parameters that determine their condition. The data derived from the sensors used to monitor them are a fundamental source for the development of mathematical models with which to predict the behaviour of conditions of the water, the sea bed and the living creatures inhabiting it. This paper is intended to explain and illustrate a design and implementation for a new multisensor monitoring buoy system. The system design is based on a number of fundamental requirements that set it apart from other recent proposals: low cost of implementation, the possibility of application in coastal shallow-water marine environments, suitable dimensions for deployment and stability of the sensor system in a shifting environment like the sea bed, and total autonomy of power supply and data recording. The buoy system has successfully performed remote monitoring of temperature and marine pressure (SBE 39 sensor), temperature (MCP9700 sensor) and atmospheric pressure (YOUNG 61302L sensor). The above requirements have been satisfactorily validated by operational trials in a marine environment. The proposed buoy sensor system thus seems to offer a broad range of applications.

## 1. Introduction

Over the last ten years, marine monitoring has become a field of major scientific interest [1]. Coastal marine systems are particularly vulnerable to the effects of human activity due to industrial, tourist and urban development. As a result, analysis of these ecosystems has become a primary concern for researchers interested in learning more about the behaviour of the marine environment. It is essential to gather information on large enough spatial and time scales to assure effective monitoring and to be able to produce solutions that as far as possible reduce the negative impact of human activity on these ecosystems.

To that end, the literature contains references to various measurement platforms which support solutions ranging from instruments integrated in on-the-spot observation systems to remote equipment, each one with a specific purpose. The observation platform may be on the surface, on board an oceanographic vessel [2,3]; it may consist of surface buoys [4,5], drifting buoys [6,7] or towed vehicles [8,9]; or it may be located on the sea bed using Autonomous Underwater Vehicles (AUVs) [10,11], Remote Operated Vehicles (ROVs) [12,13], gliders [14] or networks of underwater buoys [15], or again it may be located in space (satellites) [16]. The choice of measurement platform will be dictated mainly by the need to obtain information from an area of a given size, which implies a given density of instruments per unit of surface area, with a time frequency suited to the dynamics of the measurable parameters (temporal resolution). The choice from among the different alternatives and the selection of the instrumentation is determined, mainly, by the characteristics of the environment (sea depth, waves and tides) and the space-time coverage required. In [17] the spatial coverage of different monitoring systems versus the temporal resolution achieved with each one is showed. Some authors have solved the problems of power autonomy and collecting the recorded data by integrating sensors with surface buoys. With the information collected by those buoys, existing theoretical models can be used to achieve better spatial/temporal coverage [17].

Buoy-based coastal monitoring systems are suitable for monitoring and controlling the status of waters, as installation and maintenance are simple to perform and not very costly. Moreover, the technology used makes it possible to automate the instrumentation and sample-taking and variable adjustment of sampling frequency [18]. In this way surface sensor buoys can be deployed in a network so as to achieve a precise spatial representation of the marine variables measured. There are three possible types of sensor buoy networks depending on how the sensor nodes are linked:

1. wired buoy networks [4].
2. wireless aerial buoy networks linked by radio frequency [5], GPRS or a combination of the two [19].
3. underwater buoy networks linked by acoustic waves [15].

There are references in the literature to infrastructures that combine some of these options [20]. Of the types of network described, it is the ones using wireless communication that provide the flexibility needed for optimum data gathering and control. Thus, sensor buoys can be installed at the locations required by the instrumentation as nodes of a Wireless Sensor Network (WSN) [21,22] to make up measurement systems that are large and dense enough to provide the requisite spatial coverage.

The main advantage of marine measuring systems implemented on wireless sensor buoy networks is the possibility of remotely accessing the information recorded in the buoy from a base station. However the design, implementation and deployment of a wireless buoy network for oceanographic applications, poses new challenges different from those posed by the deployment of WSNs on land, as the marine environment imposes limits on the sensor networks, affecting their development. Despite all these drawbacks, various case studies of monitoring of marine ecosystems using WSNs can be found in the literature [18].

There are other factors that have to be taken into account when building an instrumentation platform with sensor buoys, such as the cost of marine access, and therefore the design must be such as to minimize the cost of deployment, maintenance and recovery of the buoy network. To achieve this, the sensor buoys must be equipped with energy harvesting (solar or wind-powered) systems to recharge batteries and assure energy independence. Similarly, the possibility of setting up wireless networks to link sensor buoys—a proved technology with promising results in the field of coastal oceanographic observation—considerably reduces the cost of wiring and data collection. These buoys can be designed for low energy consumption and cost. By using different topologies and taking advantage of the possibilities of WSNs, data can be gathered from buoys located several miles off the coast. At the same time, sensor buoy systems must be made of lightweight elements with a robust flotation mechanism that is free of contaminants and can withstand marine and environmental conditions. In this way we can assure the durability of the structure and minimize maintenance, which will reduce the need to access the system and hence maintenance costs.

This paper describes a low-cost wireless sensor buoy system for monitoring shallow marine environments. The sensor system is a multi-task buoy capable of incorporating sensors for shallow-water monitoring of environmental parameters and parameters on the water surface, the water column and the seabed. Tests conducted in the laboratory and in the Mediterranean Sea, have shown the viability of the system that is to be adopted for deployment of a sensor buoy network for monitoring the coast of south-east Spain as part of the Spanish National research project “Observatorio Oceanográfico Costero de la Región de Murcia” (OOCMUR). This project will address the growing need for technologies for sustainable management of the marine environment, and the need for new technological and instrumental developments for shipping and fishing enterprises. Some authors have recently tackled the development of low-cost buoy systems for purposes very like the one presented here. In [23] the authors report a low-cost marine sensor platform for monitoring in coastal and estuarine regions. The proposal is an interesting framework for addressing large area monitoring using low-cost wireless sensor networks by sacrificing accuracy at one geographic point [24] presents a low-cost sensor buoy platform that allows for temperature profiling. None of these solutions fully satisfies our requirements because either they do not allow the integration of a wide range of marine sensors, or they were not designed to be part of a WSN with RF communications. In this context, our article makes an additional contribution to the state of the art of shallow marine monitoring.

Section 2 gives a detailed description of the sensor buoy system in terms of mechanical hardware and software design. Section 3 describes the experimental scenarios in which the sensor node was tested. This is the main experimental part of the paper, and the results confirm that the hardware and software solutions proposed do indeed lead to good performance. The discussion and conclusions are contained in Sections 4 and 5, which closes the paper.

## 2. Sensor Buoy System

As noted above, the deployment of WSNs in marine environments is currently a challenge. The impact of the marine environment on the functioning of sensors, electronics and mechanical devices cannot be underestimated. For that reason, new techniques are needed to achieve this goal: addressing issues of network architecture, floating and diving sensors, protocols, security, robustness, *etc.* The existing solutions are generally ad-hoc ones as their design depends on various factors, such as the characteristics of the marine environment (deep water as opposed to shallows or coastal lagoons, climatic conditions, *etc.*), the time-scale of the deployment, the spatial scope of the deployment, and the temporal resolution of data collection. Because of all these different possible scenarios, designs have to be pre-analyzed and have to minimize the costs of deployment and maintenance of the network.

Given the purpose for which the sensor buoy system was conceived (described in detail in Section 4), a number of requirements were defined. These are:

○ *Flexibility*: the sensor buoy system must be designed so as to facilitate different configurations in terms of the physical magnitudes that can be sensed, and the timing of sampling and of storage and periodic collection of data. Moreover, the sensor buoy system should be flexible and customizable enough to accommodate heterogeneous devices and communication technologies.
○ *Energy autonomy*: the system should harvest energy from the environment in order to be able to operate without human intervention for a long time, and it should implement software for optimum management of the power necessary for the functioning of the system.
○ *Robustness and fault tolerance*: the system must have the necessary means to ensure that there is no loss of gathered data in the event of failures of communication or power supply, and that as far as possible the data collection process can continue until the connection with the system is re-established. Communications subsystems (antennas and radio modules) should be reliable and guarantee communication between sensor nodes in adverse weather conditions.
○ *Mechanical design*: components should guarantee an appropriate level of insulation and corrosion-proofing. It will be essential for the design to minimize the number of connectors used, since these are especially sensitive to corrosion in the marine environment. Moreover, the design of the buoys should facilitate access to their components for maintenance and eventual dismantling. This entails: replacing power supply systems, replacing or calibration of the sensors used, and dismantling of the sensor buoy system once the monitoring task is concluded. In addition, buoy designs should minimize the impact of their deployment on the environment that is monitored since the presence of floating buoys is usually a problem in areas with busy sea traffic.
○ *Resource optimization*: the sensor buoy system should be designed with efficient resource utilization in order to reduce the costs of manufacture, deployment, operation and maintenance.
○ *Scalability*: the buoy system should be designed to function effectively regardless of the network topology and the number of buoys that will be deployed in the monitored area.

**Figure 1.**
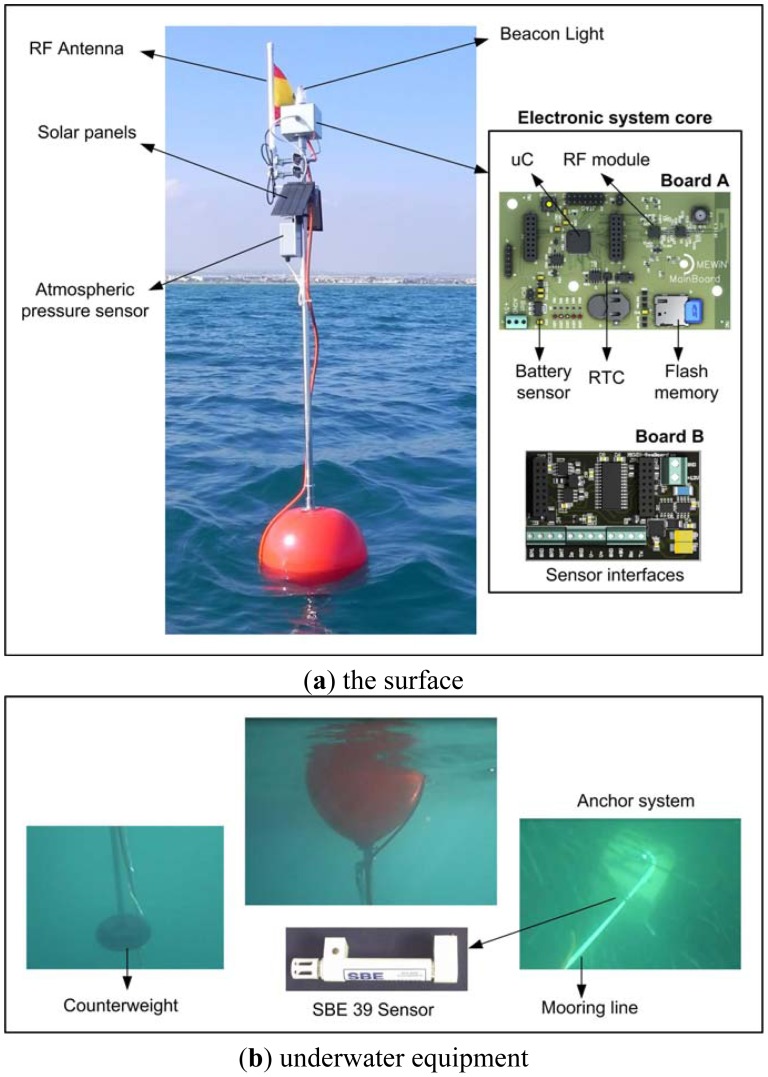
Buoy components.

Figure 1 details the elements used in the design and implementation of the sensor buoy system. As can seen, this includes a flotation device such as a buoy to keep part of the node out of the water. This out-of-the-water part includes an antenna for RF transmission, a harvesting system (solar panel) to supplement the power source, and one or more external sensors essentially to monitor meteorological data (windspeed, air temperature, atmospheric humidity, atmospheric pressure, *etc.*). The submerged part of the buoy may include several sensors, which can be placed at different depths (sensor strings), a sonde to transmit the data collected to the buoy, and finally a means of anchoring the buoy to the seabed in order to prevent it from moving (due to marine currents, wind, waves, *etc.*).

In the prototype that was designed and used in the deployment reported in this paper, the buoys are fitted with oceanographic sensors which record marine temperature and pressure. The upper part of the buoy mast, holds instrumentation to measure atmospheric pressure. The sensors and probes installed and their functional specifications are presented in Table 1. The reasons for choosing these sensors are detailed in Section 4. The hardware with which the buoy is equipped allows it to connect to any other 4–20 mA, 0–2.5 V, SDI-12, or RS-232 sensor.

The electronic system core includes: a module for RF transmissions, a power supply regulation and management system, a set of interfaces for accessing the sensors, a module for amplification, the module for conversion (analogue to digital) and multiplexing of the data read from the sensors (surface and underwater), a FLASH-type permanent read/write memory, a Real Time Clock (RTC) that performs the timer interrupts, scientific instruments (e.g., improved meteorological packages, acoustic recording packages, biological samplers, *etc.*), and a CPU (microprocessor) to centralize the whole process and implement the user-defined monitoring functions.

### 2.1. Mechanical Design

The design of the sensor buoy's mechanical structure must meet a number of requirements to assure floatability and stability at the point of measurement. These requirements include: it must be clearly visible to maritime traffic; it must be composed of non-contaminating materials that can reduce the effects of condensation; it must be stable in adverse atmospheric conditions; and the casing containing the electronic systems must be completely watertight. The mechanical design guarantees an appropriate level of insulation and corrosion-proofing since all the metal parts (structure and fixing elements) are made of marine stainless steel (316).

For rapid and efficient buoy deployment and recovery (e.g., in severe weather conditions), the structure was designed to be light and easy to assemble. However, it took a comprehensive study of wind conditions, and prevailing currents and wave velocities, to achieve a working design of the whole system. In addition, its stability was dependent on conditions such as the height at which to place the communications antenna. The marine conditions prevailing in the lagoon were determined by the results of the sea current meters installed by OOCMUR and the influence of weather conditions and season [25]. From these values, we determined the forces that would be exerted on the buoy by wind, waves and currents in unfavorable conditions, using approximate and simplified models [26]. The total surface area of the buoy at the waterline is 1.5 m^2^. The surface area of the hull is 1.37 m^2^. For simplicity's sake, we have considered the coefficients of friction of the buoy C_d_ and density φ = 1 (see Table 2). The total force (approximate) exerted by the tow point under very unfavorable conditions is 480 N.

This force value is used to calculate a mooring line suited to small anchors. A length of anchor line ranging from 3 to 5 times the depth is installed depending on the depth where the buoy is located. Because the seabed is sandy, we can expect a high coefficient of grip, and therefore it is estimated that the anchor (Hall type) will bear between 5 and 8 times its weight [25,26].

**Figure 2.**
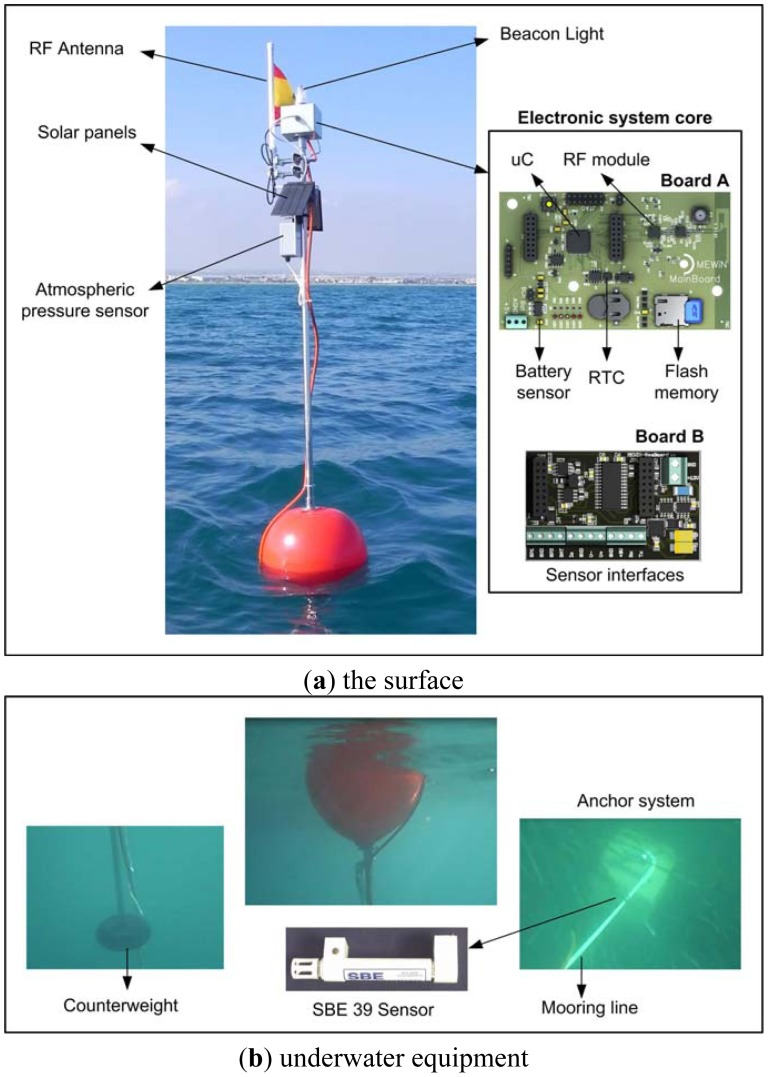
Mechanical structure of the buoy and its characteristics, including the adopted protocol, antenna and range.

The cost of the buoy had to be small in order to achieve the maximum spatial resolution with the minimum outlay. The buoy as designed is a vertical structure consisting of a stainless steel tube 3 m long and 25 mm in diameter (1) (see Figure 2). A float (2) 40 cm in diameter and 50 cm high is located exactly in the middle of the steel tube. The flotation material is polyvinyl chloride. The upper part of the buoy contains the following elements: an IP-68 watertight box 12 × 12 × 7 cm (5), an 8 dBi vertically polarized omnidirectional antenna (EnGenius NET-WL-ANT-008ON) (6) and a light-emitting beacon (4) with a twilight connection to provide night-time visibility for maritime traffic. The box contains the electronics and the storage batteries. Beneath the watertight box there are two 2.5 W solar photovoltaic panels (7) at an angle of 45° to the vertical tube. At the bottom of the structure there is a 7 kg weight (3). This weight generates a torque higher than 100 Nm (considering the buoy is tilted 30 degrees) to stabilize the buoy and keep it upright in normal sea and climatic situations. To keep the buoy in position, it is fitted with an 8 kg anchor (Hall type) attached at its centre of gravity by a rope of a length depending on the depth of the mooring. Finally, the oceanographic sensors are connected by wire to the electronics in the upper part. The total cost of the mechanical design elements (1) to (7) in Figure 2 is approximately 180 €.

### 2.2. Electronic Design

**Figure 3.**
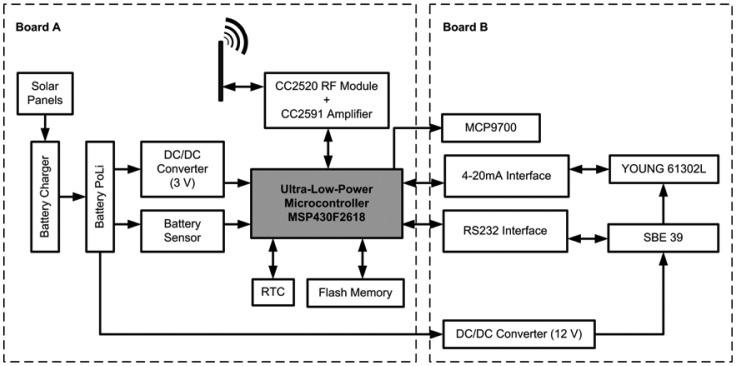
Block diagram.

The electronic systems in the buoy that perform instrumentation, recording, transmission and energy management functions were designed and developed at the Universidad Politécnica de Cartagena (Cartagena, Spain), because no commercial solution meets the required specifications (either the cost is too high, or the number of sensors that can be integrated is limited, or there is no storage capability). The authors have carried out a specific implementation of the general design they proposed in [18]. The principal components of the node are shown in block diagram of the Figure 3.

The system as developed is based on the MSP430F2618 16 bits, 92 kB Flash and 8 kB RAM Ultra-Low-Power Microcontroller from Texas Instruments (TI) and the CC2520 radio module; combined with the CC2591 device, these provide enhanced radio communication coverage. In addition, the system has been equipped with a FLASH-type read/write memory for data storage so as to avoid loss of data in the event of a loss of connection, and a RTC with which to generate timer interrupts and show the real time at which the sample was taken. The combination of these two last components is essential to avoid loss of information and to tell the exact time at which certain events occurred.

The node is powered by a 5,000 mAh lithium polymer battery (BPI LC32650 + pc) with a rated voltage of 3.7 V. The maximum output voltage of this battery is 4.2 V and is equipped with a protection circuit that prevents overcharging as well as deep discharging below 3.25 V cell voltage. The battery voltage is regulated at a constant set value of 3 V, which will be used to power each of the sensor node modules. Also, the power system has a circuit that manages the load and the switching of the 2.5 W solar panels (with a rated voltage of 8 V and a rated current of I_nominal_ = 310 mA), and it reports the voltage levels to monitor battery status. This information is available in real time, like the information supplied by the rest of the sensors. The size of the solar panels is 18 × 11 cm. The mounting angle is 45 degrees at two symmetrical opposite sides to achieve good lighting conditions even when the orientation of the buoy is not optimal. Each solar panel provides 8 V DC 310 mA in good solar conditions. It has an effectiveness of around 15%. Back plate and cover are made of epoxy fiber to improve the robustness making it suitable for outdoor applications. To make use of the sensors cited in Table 1, a RS232 connection interface is needed, and also a 4–20 mA connection interface, plus a 12 V DC/DC converter to supply the necessary power to the external sensors (SBE 39 and YOUNG 61302L). The MCP9700 temperature sensor is connected directly to an analogue microcontroller input.

The electronics that have been developed are composed of two printed circuit boards one mounted on the other (see Figure 1(A)). The first of these (Board A in Figure 3) manages the processing, communications, data storage and power management. The second one (Board B) contains the interfaces necessary to be able to use the selected sensors. In this way the system can be equipped with additional sensors or the existing ones can be modified, adding a Board B to control the new sensors. The system design is not confined to sensing of the physical magnitudes indicated earlier; it can also be extended as far as necessary, the only limiting factor being the physical space inside the box and any power restrictions that may be necessitated by an extension of the power supply system. The cost of the components needed to manufacture boards A and B comes to a total of approximately 160 €.

### 2.3. Software Design

The authors have experience in software design for sensor nodes [27] with TinyOS (the open-source operating system for low-power wireless devices) and nesC (its associated programming language). Its architecture is event-driven and based on a set of components. Such architecture simplifies the implementation of a concurrency paradigm. However, TinyOS is not compliant with the MSP430F2618 microcontroller and the CC2520 radio transceiver. Moreover, several manufactures are designing devices compliant with the high level communication protocols described in the ZigBee specification. Therefore, to allow the sensor buoy to be used with other ZigBee [28] devices and to allow a range of up to 3 km, which is sufficient coverage for the deployments, the ZigBee communication protocol was chosen. The library used in this work was Z-Stack, which provides a robust implementation of the ZigBee specification. Specifically, Z-Stack is compliant with the ZigBee 2007 (ZigBee and ZigBee PRO) specification, supporting both ZigBee and ZigBee PRO feature sets.

Together with the implementation of the communication protocols, the Z-Stack library includes an Operating System Abstraction Layer (OSAL). The OSAL implements a cooperative, round-robin task servicing loop. Each major sub-system of the Z-Stack runs as an OSAL task. Users should create at least one OSAL Task in which their application will run. This is carried out by adding their task to the task array and by calling the corresponding initialization function. There are high priority tasks, for example, the task that manages the Medium Access Control (MAC). Each task has two associated functions. One of them which is executed at the beginning of the task execution carries out the task initialization. The other one is a callback (event manager) that tests if it is necessary to process some event. Such functions are executed periodically. In short, in order to implement the set of algorithms that define the software architecture, the designer can define the events necessary for each task. Such events can be programmed to occur after a given interval. A concurrent application could thus be easily implemented using the procedure described above.

The application implemented on Z-Stack gives the sensor buoy a wide range of functionality. Sensors can be sampled with a programmable runtime frequency from 5 to 720 min, which can be adjusted remotely. Each sample is identified with the date (dd/mm/yyyy), the day of the week (1–7) and the time (hh:mm:ss) of the reading, thanks to the integrated RTC (see Figure 3). The data collected by the sensor buoy and the information about its operational status are stored in the flash memory mentioned above. The memory size is 2 GB, so that up to 31 million samples can be stored. Data stored in the flash memory can be retrieved (all the data for one day) remotely from the base station later on if the buoy goes off-line. Such a loss of signal can arise from wireless communications problems typical of the marine environment [20] (wave reflections from the sea surface, high moisture levels, movements of the sensor buoy due to waves, tides, *etc.*).

**Figure 4.**
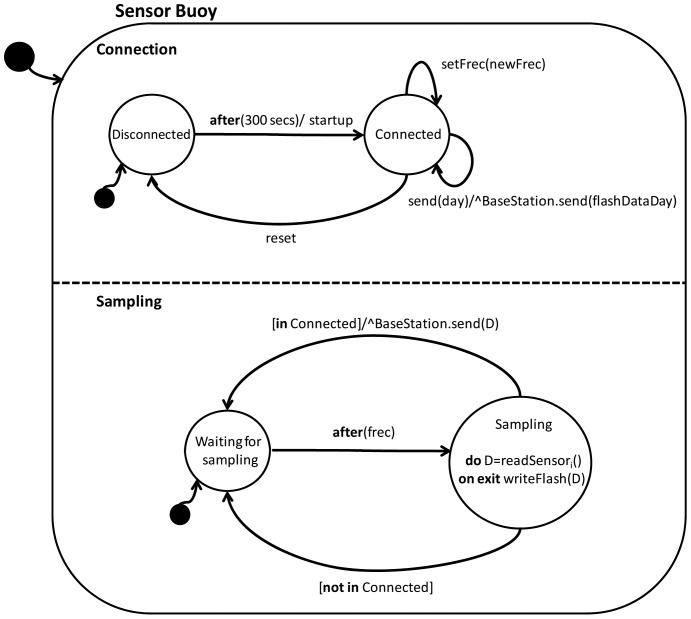
Sensor buoy function state machine.

Figure 4 shows the state machine in UML (Unified Modelling Language) notation reflecting the behaviour of the software. There are two different concurrent sub-states: Connection and Sampling. The first manages the automatic connection with the base station and supplies the stored data for a complete day if so requested by that station (message BaseStation.send (flashDataDay)). Also, the sampling frequency can be adjusted remotely (setFrec event); this will be used to model the behaviour of the second sub-state (Sampling). In this sub-state the system is on standby (state WaitingForSampling) and is activated whenever the time set (**after**) in the stored frequency (frec) is reached. At that point data are collected from each of the sensors (part **do** of the state) and data is written to the flash memory (part **on exit**). Once this is done, the system exits the Sampling sub-state and, depending on whether or not it is in Connected state (indicated by the predicate **in** Connected) it proceeds to send a message (send) with the data collected (D) to the base station. As noted in the introduction, buoy accessibility is costly, and it is therefore extremely important to optimize remote management and buoy autonomy. For example, if the sensor buoy suffers a software hangup, there is no need for physical access; it can be reset remotely from the base station.

We have been working on two lines to achieve greater buoy autonomy: connection management and optimized use of the board's electronics. As regards connection management, owing to the wireless communications problems noted above, it was necessary to consider advanced aspects relating to identification of the sensor nodes in the software implementation. This is when the buoy loses contact with the base station and reconnection has to be tried with a suitable frequency (every 300 s) rather than continuously, so as to avoid a sudden drop in the battery level. Sensor energy management is also software-driven since the sensors consume a lot of power. The system turns on the power supply a few moments before taking the measurement, takes 16 samples over a period of less than one second, turns off the sensor power supply and averages the measurements. In this way sensor energy consumption is effectively minimized. Data storage and power management together assure that the sensor system is robust, by awarding data conservation and energy saving priority over all other factors. Thus, the software detects when the battery voltage drops below a safe level (3.5 V), and duplicates the sending and sampling period to optimize the available energy.

## 3. Results and System Validation

The methodology used for validation of the sensor system was based on two experimental phases: laboratory tests and field tests. The main objective of the laboratory tests is to validate the electronics of the buoy system in terms of the target functionality and autonomy of operation. Various specific applications have been implemented to cover all possible situations. The second-phase objective is to check that the system functions properly in real maritime conditions.

### 3.1. Laboratory Tests

These tests have demonstrated that the hardware, board A and board B as described in Figure 3, functions appropriately with the selected sensors cited in Table 1. A test protocol has been defined which includes an application specifically designed to check each of the modules comprising the system. For board A we have developed a test application that records the battery voltage, loads the time of the sampling into the flash memory and sends the data to the base station by radio. This test serves to check the operation of the radio modules, RTC, memory registry and information management. Tests were run with various different configurations of CC2520 + CC2591 radio module transmission power. The maximum range achieved was 3 km with the maximum power that these modules allow (17 dBm). For board B, an application was implemented to individually test each of the board's interfaces with the sensors connected to them. After functional validation, in view of the importance of achieving the maximum possible autonomy, we conducted an exhaustive study of the sensor buoy's current consumption, connecting boards A and B and operating the system with all the sensors. The devices that had been developed were reprogrammed for low consumption as the mode best suited for operation in the field. The current measurements reported in this paper were taken with a YOKOGAWA WT210 digital wattmeter and WTVIEWER software. The data were processed after reception of the current data for the device during operation.

The sensor buoy has eight functional states, numbered as follows: (1) connection, (2) standby for messages, (3) periodic synchromizing with the base station, called data request, (4) interface enabling, (5) and (6) sensor data acquisition, (7) flash memory data storage, and (8) data transmission to the base station. The worst-case scenario was taken for average consumption.

**Figure 5.**
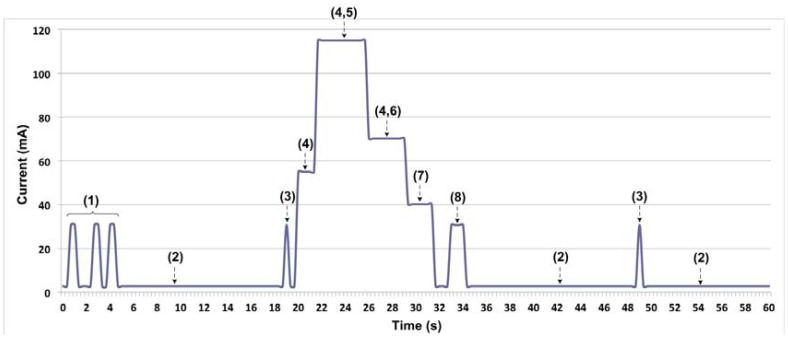
The buoy's current consumption in each of these states.

Figure 5 shows an example sequence of sensor buoy system functioning where, following the initial connection and then synchronization, the data are collected from the sensors, stored in the memory and sent to the base station. After that, the system returns to standby mode, with periodic synchronizations until the next scheduled reading process.

The ultimate aim of this study was to determine how much current the sensor buoy consumed on average, so as to relate the resulting figure directly to the capacity of the batteries and hence determine the device's autonomy. The sensor buoy's average current consumption may be determined thus:

(1) $${\bar{I}}_{\text{Total}}={\bar{I}}_{\text{Standby}}+{\bar{I}}_{\text{Connection}}+{\bar{I}}_{\text{Data Request}}+{\bar{I}}_{DC/DC}+{\bar{I}}_{\text{SBE}}+{\bar{I}}_{\text{YOUNG}\&\text{MCP}9700}+{\bar{I}}_{\text{Store Memory}}+{\bar{I}}_{\text{Send}}+{\bar{I}}_{\text{Beacon Light}}$$

Each of the current components is explained in Table 3. The worst-case scenarios were assumed in each measurement to calculate the autonomy of the sensor buoy. Thus, expression (2) shows sensor system consumption in standby mode. Equation (3) expresses the estimated average current consumed by connection to the communications network. Expression (4) refers to the periodic synchronization (15 ms every 15 s) of the sensor buoy with the base station. Expressions (5) to (9) have been calculated in the same way. The interval between samplings considered in this study was 5 min, which is the worst-case scenario as regards system consumption. The average sampling period in real deployments is 15 min. The last of the components is the average current consumed by the beacon fitted to the buoy (equation 10), which considerably raises the consumption of the system as a whole, since the buoy must show a warning light at nighttime. It is assumed that the beacon is active 12 h a day and emits 500 ms flashes every 4 s, with a consumption of 97.25 mA. As detailed in Section 3.2, the battery voltage level peaked in November, a month where the number of daylight hours is very low. For deployment of the buoy in latitudes with much more hours of darkness per day, a viability study of the harvesting system would be needed.

Regarding the addition of new sensors, the buoy's electronics are designed for low power. Our hardware design optimizes the power switch rate of the sensors to provide low overall consumption. The buoy has demonstrated that it can function autonomously for long periods. Moreover, the low-cost hardware and data management infrastructure that we have used is generic, so that the same design can be reused with multiple sensors.

(2) $${\bar{I}}_{\text{Standby}}=2.75mA$$

(3) $${\bar{I}}_{\text{Connection}}\approx 3*\frac{(30-2.75)mA\cdot 0.7s}{24*3600s}=0.66\mu A$$

(4) $${\bar{I}}_{\text{Data Request}}\approx \frac{(30-2.75)mA\cdot 0.015s}{15s}=0.027mA$$

(5) $${\bar{I}}_{\text{DCDC}}\approx \frac{(55-2.75)mA\cdot 1.7s}{300s}=0.296mA$$

(6) $${\bar{I}}_{\text{SBE}}\approx \frac{(115-2.75)mA\cdot 4.5s}{300s}=1.684mA$$

(7) $${\bar{I}}_{\text{YOUNG}\&\text{MCP}9700}\approx \frac{(70.2-2.75)mA\cdot 3.3s}{300s}=0.742mA$$

(8) $${\bar{I}}_{\text{Store Memory}}\approx \frac{(40.2-2.75)mA\cdot 2.3s}{300s}=0.287mA$$

(9) $${\bar{I}}_{\text{Send}}\approx \frac{(30-2.75)mA\cdot 1.3s}{300s}=0.121mA$$

(10) $${\bar{I}}_{\text{Beacon Light}}\approx \frac{1}{2}*\frac{(100-2.75)mA\cdot 0.5s}{4s}=6.078mA$$

(11) $$\text{Number of days}\approx \frac{5000mA\cdot h}{{\bar{I}}_{\text{Total}}mA\cdot 24h}=17.4\text{days}$$

Finally, given that the capacity of the batteries used in the system is 5,000 mAh and that Equation (1) gives an average constant current of 12 mA, the estimated autonomy without solar panels is 17.4 days (11). To guarantee energy autonomy, the system also runs two 2.5 W solar panels which assure optimum operation even under prolonged poor light conditions.

### 3.2. Field Tests

To test the buoy in marine conditions, three experiments were performed. The first field test was performed close to the port of Cartagena, which is near (less than 200 m) our laboratory. The main purpose of this test was to validate the power management system (battery charge performance and harvesting system), the communications system and the mechanical robustness of the buoy in marine conditions. Next, the second and third tests were performed on the Mar Menor lagoon in real measurement conditions.

**Figure 6.**
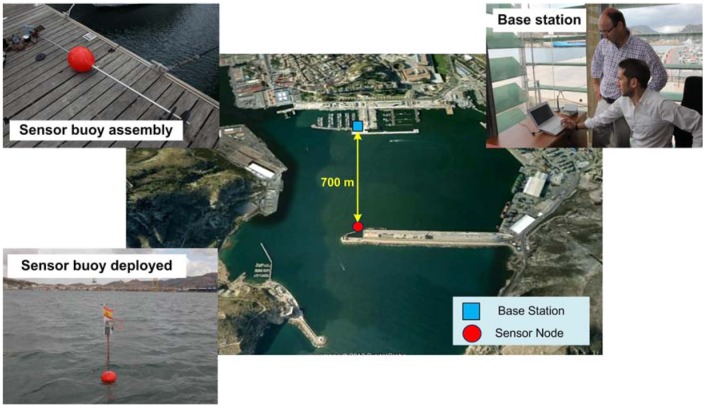
Location and deployment of tests in Cartagena Port.

The first field test consisted of a sensor buoy and a communications hardware (base station). The base station included an 5 dBi dipole antenna, 8 m above sea level. In order to choose the location of the sensor buoy, it was essential to have a direct line of sight between the antena of the base station and the sensor buoy; that the distance be right to assure good quality communication; and to locate the sensor buoy in an area with a depth of less than 7 m to use the mooring system designed. To that end, before deciding on a definitive location for the buoy in this second stage, tests were performed at various distances until an optimum location was identified on the basis of the above conditions. The distance chosen between sensor buoy and base station was 700 m (see Figure 6).

The buoy was placed at the mouth of the port of Cartagena (37°35′22.08″N, 0°58′58.84″W) and the base station was located at the offices of the Racing Club in the same port (37°35′44.92″N, 0°58′55.35″W). The complete system (buoy assembly, installation of base station and buoy deployment) took approximately 3 h to assemble. Figure 6 also shows some images of the system deployment indicating the distance from the buoy to the coast.

**Figure 7.**
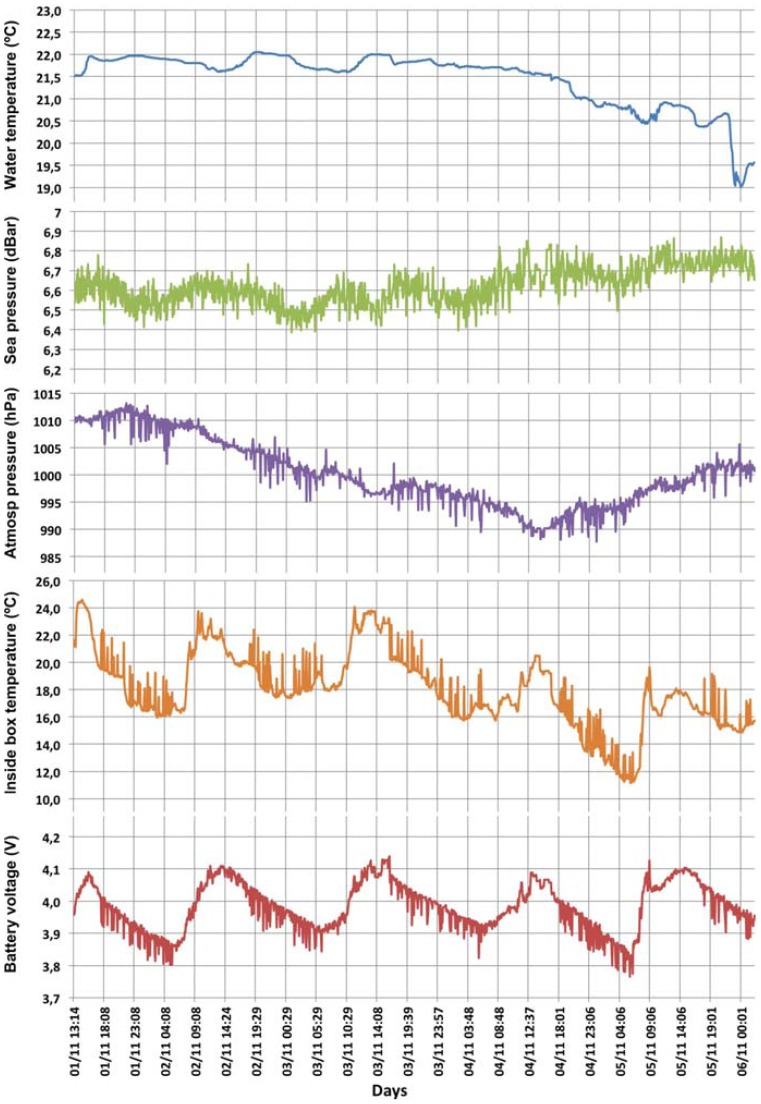
Part of the data collected by the sensor buoy.

The validation tests at sea were conducted at the location cited above and lasted two months (October–November 2011). Figure 7 shows in detail the changes in the sea temperature, the sea pressure, the atmospheric pressure, the temperature inside the box and the battery voltage recorded over 5 days during the period indicated.

As mentioned above, the battery voltage is the most important parameter when it is required to check if the power supply is properly working. The battery was installed using the maximum load voltage (4.2 V). As Figure 7 shows, the harvesting system works as expected since it completely charged the battery and achieving full load on sunny days. The battery became very depleted during the night due to the absence of sunlight and the beacon light consumption.

As regards communications between the sensor buoy and the base station, while there were some communication breakdowns caused by the factors discussed in Section 1, the functioning of the buoy system was efficient thanks to the reconnection algorithms that were implemented. The test demonstrated that the system was both robust and watertight. The system was resistant to wind and rain during the test period even under severe weather conditions, with no sign of damage to the buoy or evidence of humidity inside the box that contained the electronic components.

**Figure 8.**
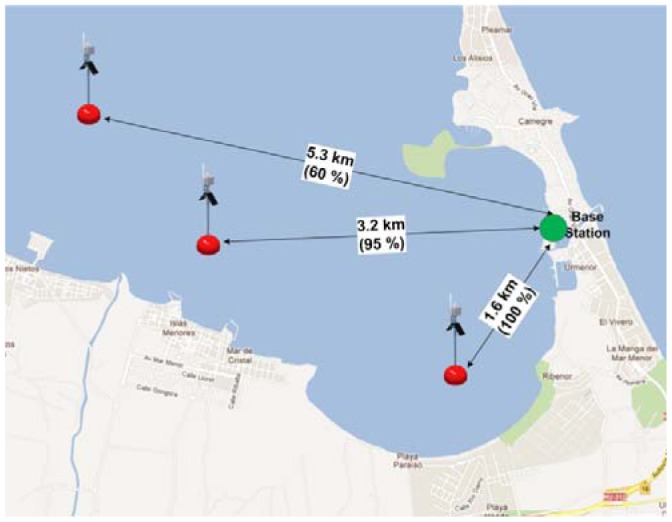
Coverage trials in the Mar Menor.

The second field test was carried out on the Mar Menor lagoon to assess the performance of the buoy in real conditions. This test was designed with the purpose of checking significant parameters such as the maximum range at which the communication between the buoys is efficient and reliable in real conditions. In order to calculate the maximum range of coverage, a preliminary survey was carried out in the deployment zone. Such range determined the minimum number of nodes needed to cover the entire monitoring area. A base station and a buoy were used in this experiment. In this case, the base station antenna was the same than the buoy one. This antenna was placed 1.5–2 m above the sea level. The sensor buoy was located at varying distances with the purpose of validating the communication coverage (see Figure 8). For each sensor buoy location, a transmission of one hundred 4-bytes was performed between the base station and the buoy. The number of packets correctly received were counted. Five attempts were carried out for each location.

Table 4 and Figure 8 show the results of this trial. They indicate the distance at which the buoy was placed and the rate of packet reception for each attempt. The weather conditions on February 16, 2010 can be listed as follows: average temperature = 9.4 °C, relative humidity = 93%, rain = 4.06 mm, wind = 8.9 km/h. Although the theoretical distances for the Fresnel zone are smaller than those obtained in the test, it would be possible to get good results with distances up to 3 km between communication devices. The system recognizes when a data frame transmission fails and it is then recorded in the internal SD card. Therefore, the data transmission can be resumed when the comunication between devices is available.

**Figure 9.**
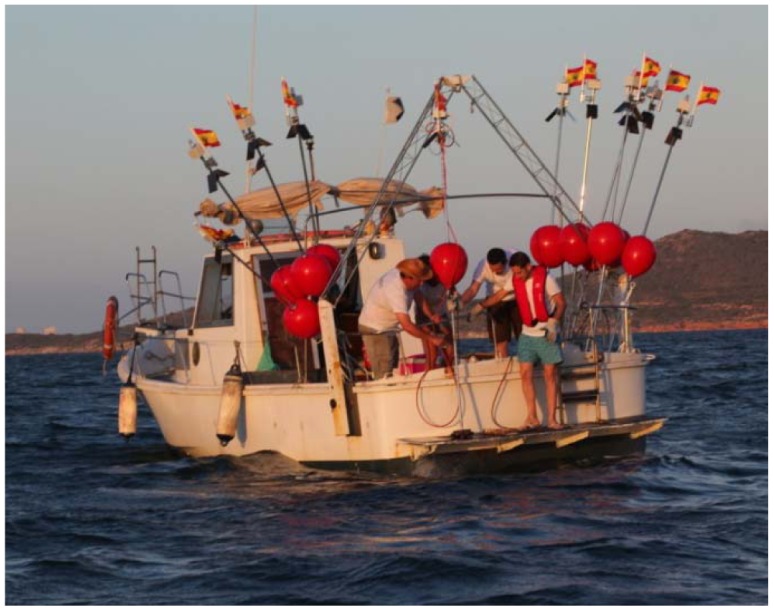
The boat used for the deployment trials in the Mar Menor.

The third field test was also carried out in real conditions on the lagoon. The purpose of this test was to check that the sensor buoys could be easily deployed, to check the performance of the communications system and to practise recovering them (see Figure 9).

Ten buoys were deployed using a star topology. All the buoys were programmed with a software application which was implemented *ad-hoc* for these trials. The sensor buoys sent a fixed message to the coordinating buoy every five minutes. These trials were carried out in 2011 in July. There is a great deal of movement of leisure craft during this month. Therefore, in order to prevent theft of equipment, the buoys were recovered the same day as soon as it was determined that the deployment worked. It was found that the buoys could be readily deployed and recovered. Because of the lightness of the structure, the buoys were easily handled by one or two persons. Deployment of the 10 buoys took approximately 3 h. The maximum radius that defined the distance between the sensor buoy and the center sink float was about half a nautical mile (800 m). All the data were successfully transmitted and collected.

## 4. Discussion

This section addresses the positive aspects and the drawbacks of the sensor system presented in this paper. The second part looks at the context in which the sensor buoy was developed.

### 4.1. Strengths and Weaknesses

Conflicts may arise between decisions on design and implementation based on the initial requirements determined by the installation conditions, in which case there will have to be tradeoffs in whatever solution is adopted. The result may not be the most functionally efficient, but it will be the best one to achieve these tradeoffs. The sensor buoy has the following strong points as shown by a detailed analysis:

1. *Low cost*. With the design and the materials used, a sensor buoy can be made for approximately 340 €; it is therefore viable for oceanographic monitoring projects where there are budgetary constraints.
2. *Ease of deployment*. Only a very simple infrastructure is necessary for its deployment in view of its small size and weight (12 kg), so that a leisure craft is sufficient.
3. *Ease of access and retrieval*. The design of the sensor buoy facilitates ready access to its components for maintenance and eventual dismantling. This essentially entails replacing power supply systems, replacing or calibration of the sensors used, and dismantling of the system once the monitoring task is concluded.
4. *Non-contaminant materials*. The design minimizes the impact of the networks deployed on the environments that are monitored.
5. *Ease of addition of new sensors*, surface or underwater.
6. *Good stability* for deployment in shallow waters.
7. Continued *improvement of communications* systems (antennas and radio modules) has been considered to make them more reliable and guarantee communication between sensor nodes in adverse weather conditions.
8. *Coverage* over a considerable distance up to 3 km, forwarding data frames when communication is active again, althoug the teoretical Fresnel zone was samaller.
9. *Energy autonomy* provided by solar panels and optimized software implementation to *reduce consumpti*on during several hours without sunlight.
10. *Insulation of components*. A design that guarantees appropriate levels of insulation and corrosion-proofing.

At the same time, we can identify the following weaknesses resulting from the compromise forced by the requirements:

1. In very adverse climatic conditions (which can occur even in shallow waters), the buoy's small dimensions may compromise its stability even with improved anchorage to the seabed.
2. Communications can be affected more readily than with a large buoy given the height of the mast and the wave attenuations and reflections that can occur as a consequence of wave movements.
3. It is easier for the buoy to be stolen given the ease of access to it.
4. The size limitation necessarily affects the equipment that can be fitted into the buoy; this restricts the number of sensors, the possibility of fitting additional electronics for pre-processing of the raw data collected, the fitting of vision systems, *etc.*

Despite these drawbacks, given the strengths described above and the scenario for which it was conceived, we believe the design and the implementation achieved are satisfactory. Following is a brief account of the purpose for which the buoy was developed, in terms of monitoring of a coastal lagoon in the Mediterranean Sea.

### 4.2. The Study Area

As detailed above, coastal ecosystems, and mainly coastal lagoons—which are among the most productive ecosystems of the planet and further play a major role in coastal fisheries as nursery and feeding grounds—are considered to be particularly vulnerable to human factors causing the erosion of marine biodiversity. Nevertheless, our understanding of the ecological factors driving the spatial and temporal variability of species and habitats in the coastal zone, which is needed for an adequate implementation of any management and enhancement measures, is strikingly poor. In this context, there is an urgent need to gain an improved understanding of the ecology of species that are important for the functioning of coastal ecosystems, linked to hydrographic processes which explain the connectivity between populations.

**Figure 10.**
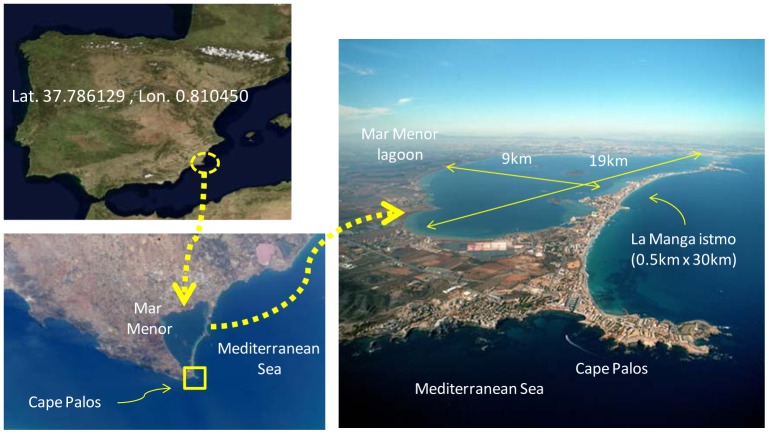
Location of the Mar Menor lagoon.

The Mar Menor coastal lagoon (as detailed in Figure 10, located on the SE coast of Spain at Latitude = 37.786129, and Longitude = 0.810450) is one of the largest in the Mediterranean, located 18 miles from cape Palos and 8 miles from cape Tiñoso, which are protected marine areas. Cape Palos in particular is a biogeographical boundary and a transitional area between the Atlantic and the Mediterranean. This lagoon is connected with the Mediterranean Sea by way of three open inlets. The main spatial dimensions of the lagoon are: N-S length 18.4 km, E-W length 8.9 km, water depth less than 7 meters; salinity range between 42–47 gr/L; pH level 7.12–8.45. Moreover, inside the lagoon there are five volcanic islands which influence the circulation patterns.

Our aim is to assess the connectivity between marine populations of species with different life strategies, to describe the genetic structure of these species on small and medium spatial scales, to study the effects of relative isolation on the genetic structure of the populations, and to evaluate the influence of lagoon environment selection in the Mar Menor lagoon. Therefore, it is particularly important to be able to monitor the main hydrodynamic, oceanographic and meteorological parameters of the Mar Menor in order to build a high-resolution numerical model capable of predicting the 3D current flows and sea elevation within the entire Mar Menor region in response to both meteorological factors and circulation in the adjacent Mediterranean Sea.

To that end we have designed, implemented and validated the low-cost sensor buoy system described above. It will be used to deploy a distributed network of sensors (as part of the OOCMUR research project) which will provide facilities and services to all international scientists interested in developing new technologies and pursuing marine research, analysing genetic samples and performing comparative studies on different spatio-temporal scales. The sensors in the sensor buoy were selected to carry out a preliminary monitoring of the study area. The objective in the first stage is to monitor the exchange of water masses between the Mar Menor coastal lagoon and the Mediterranean Sea by way of channels in La Manga. To do this, a sensor buoy will be placed on either side of the channel to sample depth variations. It is necessary to monitor the atmospheric pressure to compensate the sea depth measurement.

## 5. Conclusions

The development of sensor nodes in a marine environment is a continuous challenge today, because different requirements dictate different design solutions. The sensor buoy system described here offers a real opportunity to monitor coastal shallow-water environments. It has shown that the cost of the design and implementation of the buoy is low, that it can be integrated in a WSN, and that it can be deployed and will retain stability in aggressive and dynamic environments like the sea. It has identified the basic requirements founding the process of development of the electronic and mechanical components necessary for the assembly of a sensor set. The results have proven satisfactory in terms of performance targets, so that in the coming months it will be possible to deploy the various Mar Menor monitoring networks, in accordance with the objectives of the OOCMUR research project and gather oceanographic data over the course of this year.

## Figures and Tables

**Table 1.** Summary of devices used in the experiment.

	**Sensors**		

	**SBE39**	**YOUNG 61302L**	**MCP9700**
**Measurement**	Marine temperature and pressure	Atmospheric pressure	Temperature inside box
**Interface**	RS-232	4–20 mA	Analogue input
**Range**	T: −5 to +35 °C	500–1100 hPa	−40 °C to +125 °C
	P: 20 dBar		
**Power supply**	9–30 V_DC_	12 V_DC_	V_DD_ = 2.3 V to 5.5 V
**Consumption**	Sleep: 10 μA Per sample:	4–20 mA	6 μA
	T & time 0.018 A/sample		
	T, P, & time 0.023 A/sample		
	Continuous sampling: 15 mA		
**Manufacturer URL**	Sea-Bird Electronics http://www.seabird.com/	YOUNG http://www.youngusa.com	Microchip http://www.microchip.com/

**Table 2.** Considered oceanographic models.

**Wind**	**Sea current**	**Waves**
F_wind_ = 0.0034·V^2^_wind_·S_b_·K	F_sc_ = 2.86·S_b_·K·V^2^_sc_	F_wave_ = 0.5·C_d_·φ·S_b_V^2^_wave_
V_wind-source_ = www.aemet.es	V_c-source_ = data from OOCMUR	V_wave_ = (g·h_sea_)^0.5^

**Table 3.** Description of current components.

**Type of current**	**Description**
*Ī_standby_*	Current consumed in standby mode. This figure is subtracted from the other components to calculate their real value
*Ī_Connction_*	Current consumed in the process of connecting the sensor buoy to the base station
*Ī_Data Request_*	Current consumed in the process of periodic synchronization of the sensor buoy with the base station
*Ī_DC_*_/_*_DC_*	Current consumed with current converters in active mode
*Ī_SBE_*	Current consumed by SBE 39 sensor
*Ī_YOUNG&MCP_*_9700_	Current consumed by MCP9700 and YOUNG 61302L sensors
*Ī_Store Memory_*	Current consumed in the process of storing data in the flash memory
*Ī_Send_*	Current consumed in sending of data to the base station
*Ī_Beacon Light_*	Current consumed by light-emitting beacon

**Table 4.** Results of the communications trial.

**RANGE**	**LOCATION 1**	**LOCATION 2**	**LOCATION 3**

	**1.6 km**	**3.2 km**	**5.6 km**
Attempt 1, % pckts	100%	98%	0%
Attempt 2, % pckts	100%	93%	92%
Attempt 3, % pckts	100%	94%	82%
Attempt 4, % pckts	100%	96%	75%
Attempt 5, % pckts	100%	94%	51%
% Packets ok	100%	95%	60%
